# Detection rate of the Japan society of obstetrics and gynecology’s definition of fetal growth restriction for predicting small-for-gestational-age neonates

**DOI:** 10.1007/s10396-025-01551-2

**Published:** 2025-06-23

**Authors:** Tetsu Wakimoto, Saki Kunimoto, Ryo Yamamoto, Jun Sasahara, Keisuke Ishii

**Affiliations:** 1 Department of Maternal Fetal Medicine, Osaka Women’s and Children’s Hospital, 840 Murodocho, Izumi, Osaka 594-1101 Japan; 2 Department of Obstetrics and Gynecology, Wakimoto Clinic, Osaka, Japan

**Keywords:** Estimated fetal body weight, Fetal growth restriction, Neonates, Small for gestational age, Ultrasonography

## Abstract

**Purpose:**

To identify the ability of the definitions of fetal growth restriction (FGR) according to the Japan Society of Obstetrics and Gynecology (JSOG) and the Society for Maternal–Fetal Medicine (SMFM) to predict small-for-gestational-age (SGA) neonates.

**Methods:**

A retrospective cohort of Japanese women with singleton pregnancies who delivered at our hospital was analyzed. The primary outcome measure was the incidence of SGA neonates. The odds ratios (ORs) of SGA neonates according to the FGR definitions at 18 weeks (17–20 weeks, period 1) and 28 weeks (27–30 weeks, period 2) were calculated.

**Results:**

During periods 1 and 2, the incidence rates of SGA neonates were 7.6% and 7.7%, respectively. The ORs of the JSOG and SMFM definitions were 8.24 [95% confidence interval (CI) 4.27–14.4] and 5.88 (95% CI 3.90–8.88), respectively, during period 1 and 22.7 (95% CI 12.6–40.8) and 15.5 (95% CI 10.4–23.1), respectively, during period 2. Compared to the JSOG definition, the SMFM definition was more sensitive for predicting SGA neonates. During both periods, the positive likelihood ratio (LR +) of the JSOG definition was higher than that of the SMFM definition for predicting SGA neonates.

**Conclusion:**

The JSOG definition more strongly predicts SGA neonates and is associated with a higher LR + . The SMFM definition is highly sensitive for screening fetuses at risk for SGA status.

**Supplementary Information:**

The online version contains supplementary material available at 10.1007/s10396-025-01551-2.

## Introduction

Precise identification of fetal growth restriction (FGR) that can lead to small-for-gestational-age (SGA) neonates and adverse perinatal outcomes is an important objective in the field of perinatal medicine. Fetuses with FGR are unable to reach their biological growth potential because of placental dysfunction [1]; consequently, FGR is associated with poor perinatal outcomes [2, 3]. FGR that is not detected prenatally is associated with a risk of stillbirth that is four times higher than that of FGR detected prenatally; furthermore, antenatal FGR detection reduces the stillbirth rate by half [4]. At delivery, fetuses with FGR are more likely to be classified as SGA (defined as neonatal weight less than the 10th percentile at birth) neonates [5]. SGA neonates are vulnerable to various complications [3, 6–8], and they are at risk for long-term problems such as developmental delay, poor academic performance during childhood, and several complications at the onset of adulthood [9–12]. Therefore, FGR is considered a high-risk condition, and its diagnosis serves as a predictor of SGA neonates.

The estimated fetal body weight (EFBW) is widely determined using ultrasonography to assess fetal growth; however, it is not precise [13]. Furthermore, the EFBW is associated with an error rate of approximately > 15% [14]. Despite this limitation, the EFBW has been extensively used to define FGR in clinical practice.

The Japan Society of Obstetrics and Gynecology (JSOG) guidelines define FGR as weight less than − 1.5 standard deviations (SDs) of the local reference value (which corresponds to less than the 6.7th percentile) [15, 16]. However, an internationally accepted definition of FGR that comprises an EFBW less than the 10th percentile or abdominal circumference less than the 10th percentile is currently included in several guidelines [17–20], such as those of the Society for Maternal–Fetal Medicine (SMFM) in the United States [20, 21]. Therefore, the JSOG definition of EFBW is stricter than the SMFM definition and appears distinctive from a global perspective.

Although FGR based on the SMFM definition has been correlated with SGA status [22], whether the FGR definition based on the JSOG criteria is associated with SGA has not yet been determined. Moreover, the predictive ability of the JSOG definition of FGR for SGA neonates has not been compared to that of the SMFM definition. This study aimed to identify the characteristics of FGR based on both the JSOG and SMFM definitions as global standards and evaluate their ability to predict SGA neonates.

## Materials and methods

We conducted a retrospective cohort study of women with singleton pregnancies who presented to our institution before 14 weeks of gestation and delivered at our institution between May 1, 2015 and December 31, 2021. The estimated date of confinement was determined based on the crown–rump length [23]. The EFBW was measured at 18 weeks of gestation (range 17–20 weeks of gestation, period 1) and 28 weeks of gestation (range 27–30 weeks of gestation, period 2). Patients with fetal congenital malformations or chromosomal abnormalities were excluded. The primary outcome measure was the incidence rate of SGA neonates, which was defined as a neonatal birth weight less than the 10th percentile for gestational age according to the local reference value [5]. The secondary outcome measure was the composite adverse perinatal outcome (CAPO), which included one or more of the following events: Apgar score less than 7 at 5 min; umbilical artery (UA) pH less than 7.1; the need for intubation; respiratory distress syndrome; admission to the neonatal intensive care unit for more than 5 days; emergency cesarean delivery because of nonreassuring fetal status (NRFS) before labor onset; and fetal death or neonatal death within 7 days after birth. The NRFS included trace abnormalities of the fetal heart rate according to cardiotocography, low biophysical profile score less than 6 points or recurrent score of 6 points within 24 h, and abnormalities of the UA and ductus venosus (DV) identified during the fetal Doppler examination.

Ultrasonography was performed by certified obstetric sonographers under the supervision of experienced maternal–fetal medicine specialists. The EFBW was calculated based on the formula recommended by the Japan Society of Ultrasound in Medicine [15, 23]. FGR was defined as an EFBW less than − 1.5 SDs (6.7th percentile) of the local reference value [23]. In this study, FGR based on the SMFM definition was limited to when the EFBW was less than the 10th percentile; an abdominal circumference less than the 10th percentile was not used to define FGR. Sonographic images were obtained using the Voluson® E8 or E10 (GE Healthcare, Chicago, IL, USA) or Aplio® i700 (Canon Medical Systems Corporation, Tochigi, Japan) ultrasound machine equipped with a curved linear array transducer (2–6 MHz).

Clinical and demographic data were extracted from the hospital database and supplemented with data from the electronic medical records, including maternal age, parity, prepregnancy body mass index, smoking status, use of assisted reproductive technology, medical history, and obstetric history. Data regarding pregnancy and neonatal outcomes—including pregnancy complications, mode of delivery, neonatal birth weight, Apgar scores, UA pH, admission to the neonatal intensive care unit, respiratory distress syndrome, and the need for intubation—were also collected.

When patients were diagnosed with FGR, they underwent standardized management and serial antepartum surveillance, including a nonstress test and fetal Doppler velocimetry of the UA, middle cerebellar artery, and DV. The biophysical profile score was determined every 2 weeks until delivery or more frequently if considered necessary by the attending physician. Delivery was indicated based on the gestational age and on the EFBW when the gestational age was less than 26 weeks. Between 26 and 33 weeks of gestation, delivery was indicated if NRFS was indicated by cardiotocography results, a low biophysical profile score, or reversed flow of the a-wave of the DV observed during the fetal Doppler examination. After 34 weeks of gestation, delivery was indicated if absent or reversed diastolic flow of the UA was noted in addition to the aforementioned criteria. After 39 weeks of gestation, delivery was indicated when, in addition to the aforementioned delivery criteria, the EFBW was less than − 1.9 SDs of the local reference value (approximately less than the 3rd percentile), the pulsatility index value of the UA or DV was greater than the 95th percentile, or the pulsatility index value of the middle cerebellar artery or cerebroplacental ratio was less than the 5th percentile [23, 24]. Betamethasone was administered to pregnant women at risk for preterm delivery within 1 week before 34 weeks of gestation.

The crude odds ratios (cORs) and adjusted ORs (aORs) with 95% confidence intervals (CIs) for SGA neonates and CAPO according to the JSOG and SMFM definitions of FGR were calculated during periods 1 and 2. Variables with *P* < 0.2 in the univariate analysis were included in the multivariate analysis. The sensitivity, specificity, positive predictive value (PPV), negative predictive value, positive likelihood ratio (LR +), and negative LR (LR −) of SGA and CAPO associated with FGR were evaluated using 2 × 2 tables. Statistical analyses were conducted using EZR (Easy R) software for R (EZR is a modified version of R Commander that was designed to add structural functions that are frequently used in biostatics) [25].

This study was approved by the institutional review board of our institution (IRB No. 1632). The requirement for informed consent was waived because of the retrospective nature of this study.

## Results

A total of 3952 pregnant women were included in period 1, and a total of 3877 pregnant women were included in period 2. The maternal baseline characteristics and obstetric and neonatal outcomes are presented in Tables 1 and 2, respectively. Based on the JSOG definition, 58 (1.5%) and 57 (1.7%) neonates had FGR during periods 1 and 2, respectively. In contrast, based on the SMFM definition, 134 (3.4%) and 120 (3.1%) neonates had FGR during periods 1 and 2, respectively (Table 1). The rates of SGA neonates (primary outcome measure) and CAPO (secondary outcome measure) were 7.6% (303) and 7.3% (288), respectively, during period 1 and 7.7% (297) and 6.9% (266), respectively, during period 2 (Tables 2 and 3).

**Table 1:** Maternal characteristics at baseline

	Period 1 (18 weeks of gestation) n = 3952	Period 2 (28 weeks of gestation) n = 3877
Age (years)	34 (16–50)	34 (16–50)
Age (≥ 35 years)	1769 (44.8)	1741 (44.9)
Nulliparous	1726 (43.7)	1680 (43.3)
Prepregnancy BMI (kg/m^2^)	20.7 (14.8–52.8)	20.7 (14.8–52.8)
Prepregnancy BMI (≥ 18.5)	580 (14.7)	546 (14.7)
Smoking status	118 (3.0)	117 (3.0)
Use of assisted reproductive technology	579 (14.6)	569 (14.9)
CH	89 (2.3)	86 (2.2)
DM during pregnancy	92 (2.3)	89 (2.3)
Autoimmune disease	75 (1.9)	74 (1.9)
Hyperthyroidism (oral treatment)	31 (0.8)	20 (0.5)
Thromboembolic disease	0 (0)	0 (0)
GDM	54 (1.4)	240 (6.1)
HDP without CH	1 (0.03)	4 (0.1)
FGR based on the JSOG definition	58 (1.5)	57 (1.7)
FGR based on the SMFM definition	135 (3.4)	120 (3.1)

Data are presented as n (%) or median (minimum–maximum)

*BMI* body mass index, *CH* chronic hypertension, *DM* diabetes mellitus, *GDM* gestational diabetes mellitus, *HDP* hypertensive disorders of pregnancy, *FGR* fetal growth restriction, *JSOG* Japan Society of Obstetrics and Gynecology, *SMFM* Society for Maternal–Fetal Medicine

**Table 2:** Obstetric and neonatal outcomes

	Period 1 (18 weeks of gestation) n = 3952	Period 2 (28 weeks of gestation) n = 3877
Gestational age at delivery (weeks)	39.3 (22.1–42.0)	39.3 (27.5–42.0)
Preterm birth	219 (5.5)	188 (5.0)
Mode of delivery		
Vaginal delivery	2967 (75.1)	2912 (75.1)
Elective cesarean delivery	584 (14.8)	576 (14.9)
Emergency cesarean delivery	401 (10.1)	389 (10.0)
Preterm birth because of fetal reasons	23 (0.6)	19 (0.5)
Abruption	16 (0.4)	14 (0.3)
Cesarean delivery before labor onset attributable to NRFS	26 (0.7)	22 (0.6)
Miscarriage before 22 weeks	3 (0.07)	-
Perinatal death	7 (0.17)	2 (0.05)
Neonatal birth weight (g)	3040 (424–4776)†	3042 (882–4776)§
SGA neonate	303 (7.6)‡	297 (7.7)¶
Neonatal sex (male)	2043 (51.8)†	1922 (51.7)§

^†^n = 3943, stillbirths were excluded

^‡^n = 3941, stillbirths and neonates delivered after 42 weeks of gestation were excluded

^§^n = 3875, stillbirths were excluded

^¶^n = 3873, stillbirths and neonates delivered after 42 weeks of gestation were excluded

Data are shown as *n* (%) or median (minimum–maximum)

*NRFS* nonreassuring fetal status, *SGA* small for gestational age

**Table 3:** Patient outcomes

	Period 1 (18 weeks of gestation) n = 3952	Period 2 (28 weeks of gestation) n = 3877
CAPO^†^	288 (7.3)	266 (6.9)
Perinatal death	10 (0.3)	2 (0.05)
Cesarean delivery before labor onset attributable to NRFS	26 (0.7)	23 (0.6)
UA pH < 7.1	27 (0.7)	26 (0.7)
Apgar score < 7 at 5 min	36 (0.9)	24 (0.6)
RDS	42 (1.1)	34 (0.9)
Intubation	75 (1.9)	64 (1.7)
NICU admission	221 (5.6)	208 (5.4)

Parameters were not available for all patients

^†^CAPO was defined as at least one of the following: perinatal death, cesarean delivery before labor onset attributable to NRFS, UA pH < 7.1, 5-min Apgar score < 7, RDS, intubation, and NICU admission

*CAPO* composite adverse perinatal outcome, *UA* umbilical artery, *NICU* neonatal intensive care unit, *NRFS* nonreassuring fetal status, *RDS* respiratory distress syndrome

**Fig. 1:**
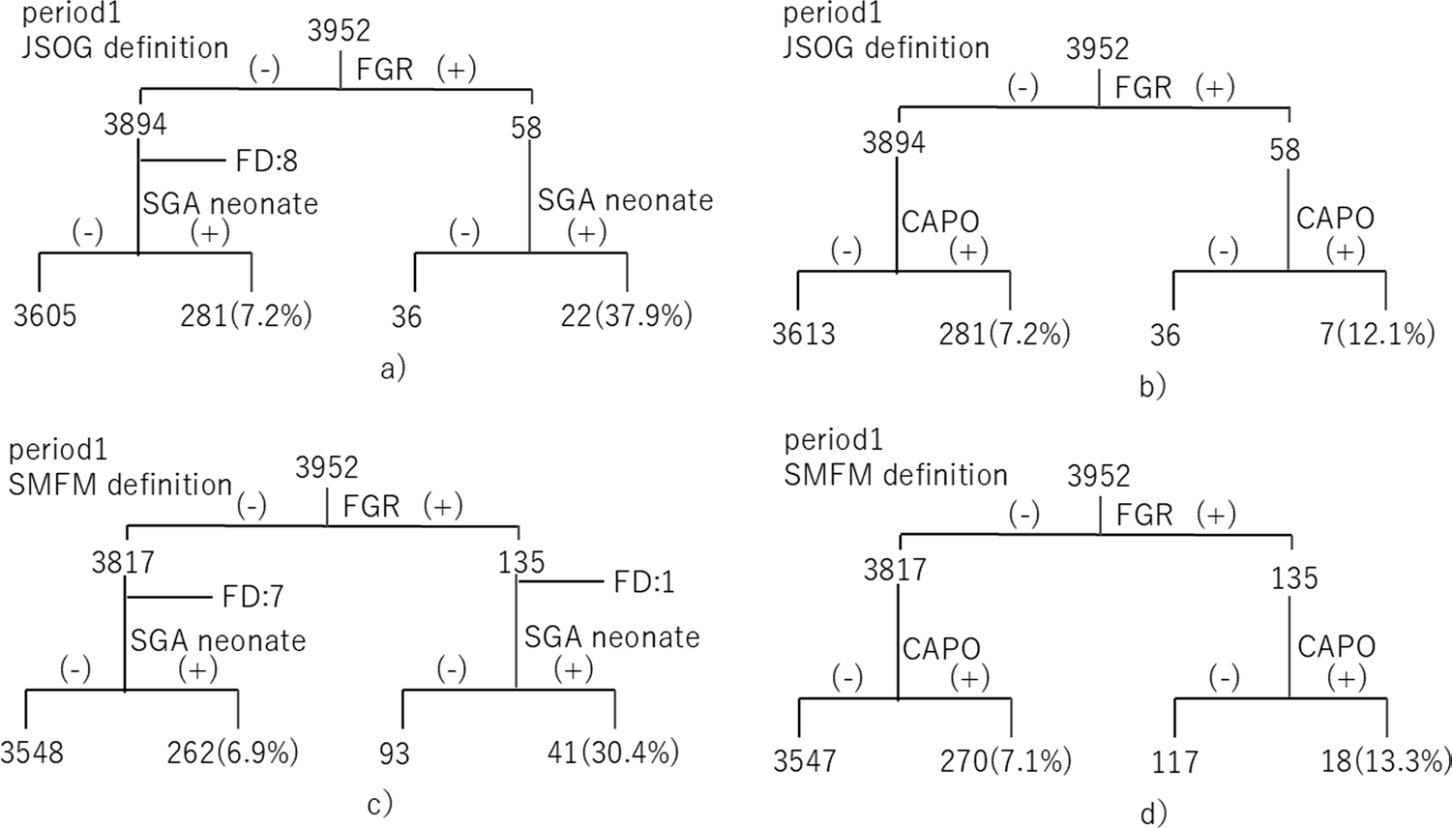
SGA neonates and CAPO during period 1. **a** SGA neonates according to the JSOG definition. **b** CAPO according to the JSOG definition. **c** SGA neonates according to the SMFM definition. **d** CAPO according to the SMFM definition. *CAPO* composite adverse perinatal outcome, *FGR* fetal growth restriction, *JSOG* Japan Society of Obstetrics and Gynecology, *SGA* small for gestational age, *SMFM* Society for Maternal–Fetal Medicine

**Fig. 2:**
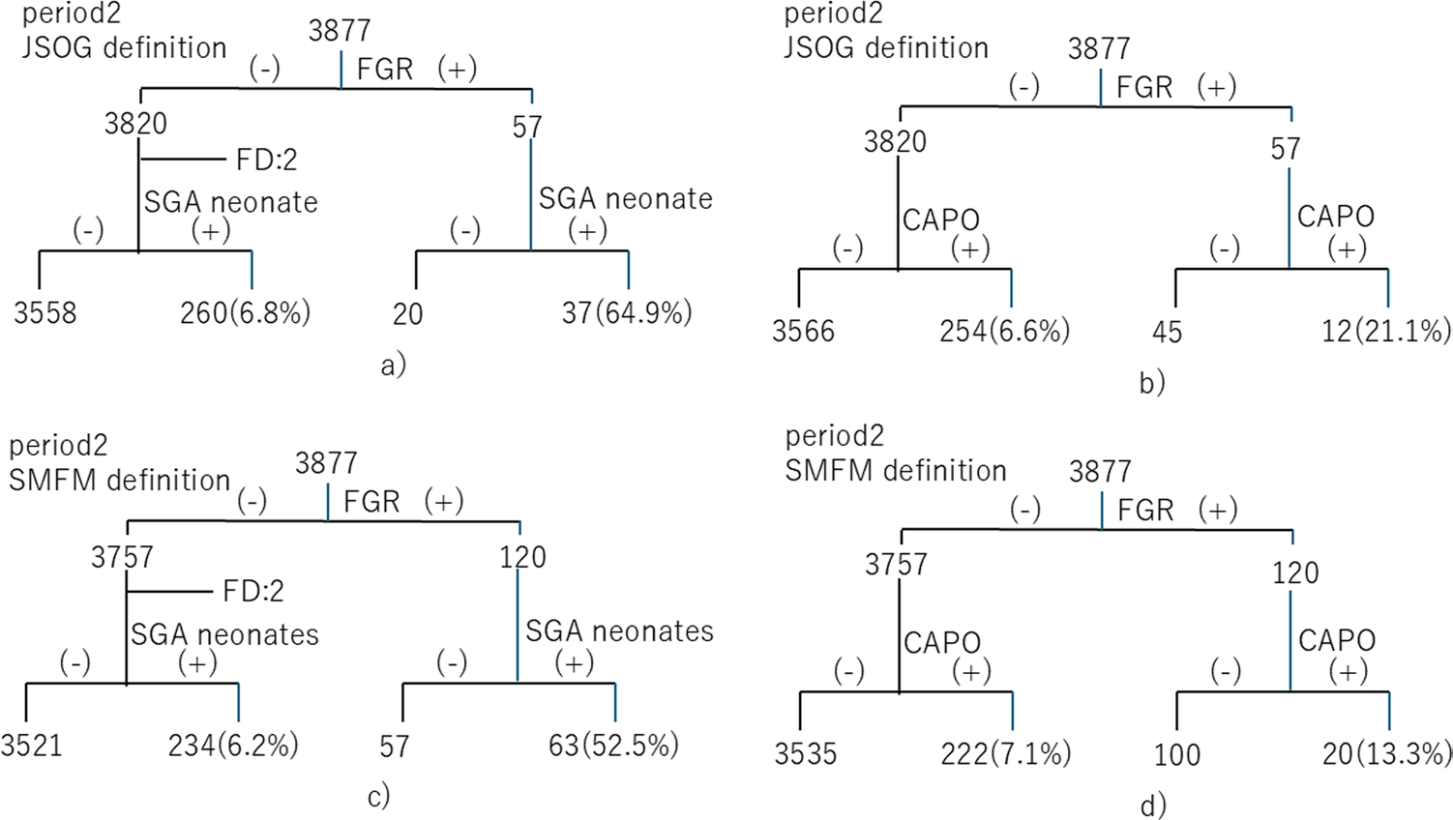
SGA neonates and CAPO during period 2. **a** SGA neonates according to the JSOG definition. **b** CAPO according to the JSOG definition. **c** SGA neonates according to the SMFM definition. **d** CAPO according to the SMFM definition. *CAPO* composite adverse perinatal outcome, *FGR* fetal growth restriction, *JSOG* Japan Society of Obstetrics and Gynecology, *SGA* small for gestational age, *SMFM* Society for Maternal–Fetal Medicine

The rates of SGA neonates and CAPO associated with cases with and without FGR during periods 1 and 2 are shown in Figs. 1 and 2. During period 1, among the 58 fetuses with FGR diagnosed based on the JSOG definition, 22 (37.9%) were classified as SGA neonates at birth and seven (12.1%) experienced CAPO at birth. Among the 3894 fetuses who were not considered to have FGR according to the JSOG definition (excluding eight cases of fetal death), 281 (7.2%) were classified as SGA neonates and 281 (7.2%) experienced CAPO (including cases of fetal death). Additionally, during period 1, among the 135 fetuses diagnosed with FGR based on the SMFM definition (excluding one case of fetal death), 41 (30.4%) were classified as SGA neonates at birth and 18 (13.3%) experienced CAPO (including cases of fetal death). Among the 3817 fetuses who were not considered to have FGR based on the SMFM definition (excluding seven cases of fetal death), 262 (6.9%) were classified as SGA neonates and 270 (7.1%) experienced CAPO (including cases of fetal death).

During period 2, among the 57 fetuses with FGR diagnosed based on the JSOG definition, 37 (64.9%) were classified as SGA neonates at birth and 12 (21.1%) experienced CAPO. Among the 3820 fetuses who were not considered to have FGR based on the JSOG definition (excluding two cases of fetal death), 260 (6.8%) were classified as SGA neonates and 254 (6.6%) experienced CAPO (including cases of fetal death). Additionally, during period 2, among the 120 fetuses diagnosed with FGR based on the SMFM definition, 63 (52.5%) were classified as SGA neonates at birth and 20 (16.6%) experienced CAPO (including cases of fetal death). Among the 3757 fetuses who were not considered to have FGR based on the SMFM definition (excluding two cases of fetal death), 234 (6.2%) were classified as SGA neonates and 222 (5.9%) experienced CAPO (including cases of fetal death).

Based on the results of logistic regression analysis, the aORs of SGA neonates based on the JSOG definition of FGR during periods 1 and 2 were 8.24 (95% CI: 4.27–14.4, *P* < 0.01) and 22.7 (95% CI 12.6–40.8, *P* < 0.01), respectively (Table 4a, b). Furthermore, the aORs of SGA neonates based on the SMFM definition of FGR during periods 1 and 2 were 5.88 (95% CI 3.90–8.88, *P* < 0.01) and 15.5 (95% CI: 10.4–23.1, *P* < 0.01), respectively (Table 4c, d). The aORs of SGA neonates based on risk factors other than FGR defined by the JSOG and SMFM definitions are shown in Supplementary Table 1.

**Table 4:** SGA neonates and CAPO based on the JSOG and SMFM definitions of FGR

	cOR (95% CI)	*P*	aOR (95% CI)	*P*
*a) SGA neonates and CAPO based on the JSOG definition of FGR at 18 weeks of gestation (period 1)*				
SGA neonates	7.84 (4.55–13.50)	< 0.01	8.24 (4.27–14.4)	< 0.01
CAPO	1.76 (0.79–3.92)	0.16	1.98 (0.86–4.53)	0.11
*b) SGA neonates and CAPO based on the JSOG definition of FGR at 28 weeks of gestation (period 2)*				
SGA neonates	25.8 (14.6–45.7)	< 0.01	22.7 (12.6–40.8)	< 0.01
CAPO	4.56 (2.41–8.60)	< 0.01	3.94 (2.00–7.74)	< 0.01
*c) SGA neonates and CAPO based on the SMFM definition of FGR at 18 weeks of gestation (period 1)*				
	cOR (95% CI)	*P*	aOR (95% CI)	*P*
SGA neonates	5.97 (4.05–8.80)	< 0.01	5.88 (3.90–8.88)	< 0.01
CAPO	2.02 (1.21–3.37)	< 0.01	1.76 (0.98–3.17)	0.06
*d) SGA neonates and CAPO based on the SMFM definition of FGR at 28 weeks of gestation (period 2)*				
SGA neonates	16.6 (11.3–24.5)	< 0.01	15.5 (10.4–23.1)	< 0.01
CAPO	2.85 (1.74–4.69)	< 0.01	2.60 (1.54–4.39)	< 0.01

*aOR* adjusted odds ratio, *CAPO* composite adverse perinatal outcome, *CI* confidence interval, *cOR* crude odds ratio, *FGR* fetal growth restriction, *JSOG* Japan Society of Obstetrics and Gynecology, *SGA* small for gestational age, *SMFM* Society for Maternal–Fetal Medicine

The JSOG definition of FGR was not associated with the CAPO incidence of SGA neonates during period 1 (aOR: 1.98, 95% CI 0.86–4.53, *P* = 0.11) (Table 4a); however, it was independently associated with the CAPO incidence during period 2 (aOR: 3.94, 95% CI 2.00–7.74, *P* < 0.01) (Table 4b). Similarly, the SMFM definition of FGR was not associated with the CAPO incidence during period 1 (aOR: 1.76, 95% CI 0.98–3.17, *P* = 0.06) (Table 4c); however, it was independently associated with the CAPO incidence during period 2 (aOR: 2.60, 95% CI 1.54–4.39, *P* < 0.01) (Table 4d). The aORs of CAPO associated with factors other than the JSOG and SMFM definitions of FGR are shown in Supplementary Table 2.

The diagnostic performance of the JSOG definition of FGR and that of the SMFM definition of FGR for SGA neonates and CAPO were compared (Table 5). The JSOG definition of FGR had lower sensitivity for predicting SGA neonates compared to that of the SMFM definition during both periods; however, the PPV of the JSOG definition for SGA neonates was higher than that of the SMFM definition during both periods. Additionally, the LR + of the JSOG definition for SGA neonates was higher than that of the SMFM definition (LR + with FGR based on the JSOG definition: 7.34 during period 1 and 22.9 during period 2, LR + with FGR based on the SMFM definition: 5.30 during period 1 and 13.3 during period 2).

**Table 5:** Diagnostic performance of the FGR definitions for SGA neonates and CAPO at 18 weeks (period 1) and 28 weeks (period 2) of gestation

	SE (%)	SP (%)	PPV (%)	NPV (%)	LR +	LR −
*SGA neonates at 18 weeks of gestation (period 1)*						
FGR based on the JSOG definition	7.3	99.0	37.9	92.8	7.34	0.94
FGR based on the SMFM definition	13.5	97.4	30.6	93.1	5.30	0.89
*SGA neonates at 28 weeks of gestation (period 2)*						
FGR based on the JSOG definition	12.5	99.4	64.9	93.2	22.9	0.88
FGR based on the SMFM definition	21.2	98.4	52.5	93.8	13.3	0.80
*CAPO at 18 weeks of gestation (period 1)*						
FGR based on the JSOG definition	2.4	98.6	12.1	92.8	1.75	0.99
FGR based on the SMFM definition	6.2	96.8	13.3	92.9	1.96	0.97
*CAPO at 28 weeks of gestation (period 2)*						
FGR based on the JSOG definition	5.3	98.8	23.6	93.6	4.34	0.96
FGR based on the SMFM definition	8.1	97.2	17.2	93.7	2.94	0.95

*CAPO* composite adverse perinatal outcome; FGR, fetal growth restriction, *JSOG* Japan Society of Obstetrics and Gynecology, *LR + * positive likelihood ratio, *LR − * negative likelihood ratio, *NPV* negative predictive value, *PPV* positive predictive value, *SE* sensitivity, *SGA* small for gestational age, *SMFM* Society for Maternal–Fetal Medicine, *SP* specificity

Regarding CAPO, the JSOG definition of FGR had lower sensitivity than that of the SMFM definition during both periods. The PPV of the JSOG definition of FGR was higher than that of the SMFM definition during period 1, but it was lower than that of the SMFM definition during period 2. Although the LR + was approximately the same with both definitions during period 1 (JSOG: 1.75, SMFM: 1.96), it was lower with the SMFM definition during period 2 (JSOG: 4.34, SMFM: 2.94).

## Discussion

This study was conducted at a single institution that performed standardized perinatal management. FGR, which was diagnosed based on both the JSOG and SMFM definitions at 18 and 28 weeks of gestation, was significantly associated with the occurrences of SGA neonates and CAPO. Therefore, FGR diagnoses at these two time points are clinically valuable.

According to the International Society of Ultrasound in Obstetrics and Gynecology (ISUOG) guidelines, a routine mid-trimester fetal examination should be performed between 18 and 22 weeks of gestation. Additionally, depending on the patient’s risk, an ultrasound examination should be performed at 32 or 36 weeks of gestation to detect FGR [26]. The American Congress of Obstetrics and Gynecology and American Institute of Ultrasound in Medicine recommend fetal examinations between 18 and 22 weeks of gestation and during the third trimester [3, 27]. Furthermore, the JSOG guidelines recommend fetal examinations between 18 and 20 weeks of gestation and between 28 and 30 weeks of gestation [16]. Therefore, it is reasonable to analyze data at 18 and 28 weeks of gestation. Although several studies have shown that the EFBW at 36 weeks of gestation is a better predictor of SGA and CAPO than that at 32 weeks of gestation [28–30], the present study also indicated that FGR at 18 weeks of gestation and FGR at 28 weeks of gestation are associated with SGA and CAPO. Therefore, careful management is necessary when FGR is diagnosed at these points.

In this study, the incidence of FGR diagnosed based on both definitions was lower than anticipated. The limited number of FGR cases observed during this study can be attributed to the exclusion of fetuses with congenital abnormalities and the somewhat small sample size. Additionally, it has been speculated that the local Japanese reference standard [23], which was established in the 1990s based on the data of healthy pregnant women at that time [23, 31], may not accurately reflect the contemporary Japanese population.

Our results indicate that the sensitivity of the JSOG definition of FGR for predicting SGA neonates was lower than that of the SMFM definition; however, the PPV of the JSOG definition of FGR for predicting SGA neonates was higher than that of the SMFM definition. Additionally, the LR + for SGA neonates according to the JSOG definition was higher than that according to the SMFM definition. These findings suggest that the JSOG definition is superior to the SMFM definition for prenatally predicting SGA neonates. The sensitivity of the SMFM definition of FGR for predicting SGA neonates was higher than that of the JSOG definition. Our results suggest that when performing screening tests to identify fetuses at risk for SGA status, the SMFM definition of FGR may be preferable to the JSOG definition.

Because of the low incidence of CAPO with both definitions, it was difficult to compare the diagnostic performance of these definitions in this regard during this study. However, it is still important to identify FGR during these periods because severe FGR and insufficient antenatal identification of FGR can lead to intrauterine fetal death [32]. If FGR is diagnosed, then more intensive management is required. The ISUOG guidelines advocate the 2016 diagnostic criteria for FGR to appropriately identify fetuses at risk for perinatal adverse events [1]. Distinctive features of the FGR definition based on the ISUOG guidelines include fetal Doppler evaluations as well as fetal biometry. Similarly, the International Federation of Gynecology and Obstetrics announced that Doppler assessments are integral to the diagnosis and management of FGR [33]. Based on the results of previous studies [22, 34], the EFBW alone may not be sufficient for predicting CAPO. The predictive ability of the EFBW for CAPO is presumably improved by performing fetal Doppler velocimetry of the UA, middle cerebellar artery, and DV [35, 36]. To appropriately identify fetuses at high risk for SGA status throughout pregnancy, an FGR management system should be established after its initial diagnosis based solely on the EFBW.

Neonatal management of a newborn with SGA status who was not diagnosed with FGR before delivery can be problematic. The probability of a newborn with SGA status without a diagnosis of FGR before birth is expressed as (100 − NPV)%. During this study, these probability rates were 7.2% according to the JSOG definition and 6.9% according to the SMFM definition during period 1; additionally, they were 6.8% according to the JSOG definition and 6.2% according to the SMFM definition during period 2. Therefore, the false-negative probability rates associated with the JSOG definition are 1.04 times higher during period 1 and 1.1 times higher during period 2 compared to those associated with the SMFM definition.

However, the false-positive probability rate of FGR is expressed as (100 − PPV)%. During this study, these probability rates were 62.1% according to the JSOG definition and 69.4% according to the SMFM definition during period 1; additionally, they were 35.1% according to the JSOG definition and 47.5% according to the SMFM definition during period 2. Therefore, the false-positive probability rates associated with the SMFM definition were 1.12 times higher during period 1 and 1.35 times higher during period 2 compared to those associated with the JSOG definition.

Diagnosing FGR based on the SMFM definition and managing perinatal care accordingly may reduce the risk of missing fetuses at high risk for SGA status at delivery; however, excessive medical intervention for some healthy fetuses is concerning.

Because of the false-positive rates associated with the SMFM definition, it is important to identify fetuses who are actually at risk for SGA status and those who require intensive perinatal management using indicators other than the EFBW. Although such indicators have not yet been established, the aforementioned ISUOG FGR guidelines, which were created by an international group of experts using the Delphi method, can be used as a reference [37]. The ISUOG FGR guidelines indicate that the degree of fetal smallness and fetal Doppler evaluation results can be used as diagnostic criteria for FGR. Additionally, other various factors associated with the risk of FGR should be stratified, and optimal management methods for individual patients that are tailored to each risk factor should be established. Therefore, further studies are warranted.

The strengths of this study are multifaceted. First, this study was conducted at a single institution that performs standardized perinatal management for FGR, thus ensuring consistent care. Second, all estimated dates of confinement were determined based on the crown–rump length measured during the first trimester, thus providing accurate gestational dating. Third, this is the first study to clarify the association between FGR, as defined by JSOG, and the incidence rate of SGA neonates. Finally, it compared the effectiveness of the JSOG and SMFM definitions of FGR to predict perinatal outcomes.

However, this study also had some limitations. First, it was a retrospective study. Second, the sample size was small. Third, the formula for the EFBW used by the JSOG differed from that used by the SMFM. Fourth, the SMFM definition of FGR includes abdominal circumference criteria, whereas the JSOG definition does not. Consequently, we could not perform direct comparisons of the JSOG and SMFM definitions. Instead, we compared the incidences of SGA neonates and CAPO according to the EFBW. Finally, the EFBW was measured at specified times during this study. However, different results were observed when the relationship between the EFBW measured immediately before delivery and the outcomes was investigated.

## Conclusion

Both definitions of FGR were related to the incidence rates of SGA neonates during both periods. However, the JSOG definition affirmed the presence of SGA status based on the higher LR + . Because of its higher sensitivity, the SMFM definition was more effective than the JSOG definition when screening fetuses at risk for SGA status.

## Supplementary Information

Below is the link to the electronic supplementary material.

Supplementary file1 (DOCX 24 KB)

## Data Availability

The data in this manuscript can be made available upon request.
