# Supplementary material for: Detection rate of the Japan society of obstetrics and gynecology’s definition of fetal growth restriction for predicting small-for-gestational-age neonates
Source: J Med Ultrason (2001). 2025 Jun 23;52(4):395–403. doi: 10.1007/s10396-025-01551-2 (PMC12999695; doi:10.1007/s10396-025-01551-2)
Supplement: Supplementary file 1 — Supplementary file1 (DOCX 24 KB) [file 10396_2025_1551_MOESM1_ESM.docx]

Supplementary Table 1. Odds ratios for SGA neonates based on the JSOG and SMFM definitions of FGR

a) Odds ratios for SGA neonates based on the JSOG definition of FGR at 18 weeks of gestation (period 1)

|  | cOR (95% CI) | *P* | aOR (95% CI) | *P* |
| --- | --- | --- | --- | --- |
| Prepregnancy BMI <18.5 kg/m^2^ | 1.97 (1.48–2.62) | <0.01 | 2.08 (1.54–2.79) | <0.01 |
| Smoking status | 3.25 (2.04–5.17) | <0.01 | 2.94 (1.80–4.81) | <0.01 |
| Use of assisted reproductive technology | 0.60 (0.40–0.88) | <0.01 | 0.67 (0.44–1.02) | 0.06 |
| CH | 3.18 (1.87–5.40) | <0.01 | 3.28 (1.83–5.87) | <0.01 |
| Hyperthyroidism (oral treatment) | 3.04(1.23–7.51) | <0.05 | 3.31 (1.32-8.31) | 0.05 |
| Autoimmune disease | 1.91 (1.00–3.75) | 0.06 | 1.83 (0.88–3.79) | 0.11 |
| FGR based on the  JSOG definition | 7.84 (4.55–13.50) | <0.01 | 8.24 (4.27–14.4) | <0.01 |

b) Odds ratios for SGA neonates based on the JSOG definition of FGR at 28 weeks of gestation (period 2)

|  | cOR (95% CI) | *P* | aOR (95% CI) | *P* |
| --- | --- | --- | --- | --- |
| Smoking status | 3.24 (2.02–5.22) | <0.01 | 3.16 (1.91–5.21) | <0.01 |
| Use of assisted reproductive technology | 0.58 (0.39–0.87) | <0.01 | 0.64 (0.42–0.98) | <0.05 |
| CH | 3.07 (1.75–5.37) | <0.01 | 2.64 (1.42–4.90) | <0.01 |
| Hyperthyroidism  (oral treatment) | 4.34 (1.55–12.1) | <0.01 | 4.60 (1.62–13.1) | <0.01 |
| Autoimmune disease | 1.89 (0.93–3.85) | 0.08 | 2.19 (1.06–4.52) | <0.05 |
| HDP without CH | 2.25 (1.59–3.19) | <0.05 | 2.26 (1.55–3.29) | <0.01 |
| FGR based on the  JSOG definition | 25.8 (14.6–45.7) | <0.01 | 22.7 (12.6–40.8) | <0.01 |

c) Odds ratios for SGA neonates based on the SMFM definition of FGR at 18 weeks of gestation (period 1)

|  | cOR (95% CI) | *P* | aOR (95% CI) | *P* |
| --- | --- | --- | --- | --- |
| Prepregnancy BMI <18.5 kg/m^2^ | 1.97 (1.48–2.62) | <0.01 | 2.02 (1.50–2.72) | <0.01 |
| Smoking status | 3.25 (2.04–5.17) | <0.01 | 3.16 (1.94–5.16) | <0.01 |
| Use of assisted reproductive technology | 0.60 (0.40–0.88) | <0.01 | 0.71 (0.46–1.08) | 0.11 |
| CH | 3.18 (1.87–5.40) | <0.01 | 3.24 (1.81–5.83) | <0.01 |
| Hyperthyroidism (oral treatment) | 3.04 (1.23–7.51) | <0.05 | 3.47 (1.38-8.74) | <0.01 |
| Autoimmune disease | 1.91 (1.00–3.75) | 0.06 | 1.95 (0.94–4.05) | 0.07 |
| FGR based on the  SMFM definition | 5.97 (4.05–8.80) | <0.01 | 5.88 (3.90–8.88) | <0.01 |

d) Odds ratios for SGA neonates based on the SMFM definition of FGR at 28 weeks of gestation (period 2)

|  | cOR (95% CI) | *P* | aOR (95% CI) | *P* |
| --- | --- | --- | --- | --- |
| Smoking status | 3.24 (2.02–5.22) | <0.01 | 2.70 (1.59–4.60) | <0.01 |
| Use of assisted reproductive technology | 0.58 (0.39–0.87) | <0.01 | 0.67 (0.44–1.03) | 0.07 |
| CH | 3.07 (1.75–5.37) | <0.01 | 2.76 (1.48–5.14) | <0.01 |
| Hyperthyroidism  (oral treatment) | 4.34 (1.55–12.1) | <0.01 | 5.03 (1.76–14.4) | <0.01 |
| Autoimmune disease | 1.89 (0.93–3.85) | 0.08 | 2.41 (1.17–4.97) | <0.05 |
| HDP without CH | 2.25 (1.59–3.19) | <0.05 | 2.28 (1.56–3.34) | <0.01 |
| FGR based on the  SMFM definition | 16.6 (11.3–24.5) | <0.01 | 15.5 (10.4–23.1) | <0.01 |

aOR, adjusted odds ratio; BMI, body mass index; CH, chronic hypertension; CI, confidence interval; cOR, crude odds ratio; FGR, fetal growth restriction; HDP; hypertensive disorders of pregnancy; JSOG; Japan Society of Obstetrics and Gynecology; SGA, small for gestational age.

Supplementary Table 2. Odds ratios for CAPO based on the JSOG and SMFM definitions of FGR

a) Odds ratios for CAPO based on the JSOG definition of FGR at 18 weeks of gestation (period 1)

|  | cOR (95% CI) | *P* | aOR (95% CI) | *P* |
| --- | --- | --- | --- | --- |
| Prepregnancy BMI <18.5 kg/m^2^ | 0.73 (0.49–1.11) | 0.14 | 0.85 (0.56–1.28) | 0.43 |
| Smoking during pregnancy | 1.90 (1.09–3.31) | <0.05 | 1.54 (0.81–2.90) | 0.18 |
| Use of assisted reproductive technology | 1.25 (0.91–1.71) | 0.18 | 1.25 (0.87–1.79) | 0.23 |
| CH | 5.35 (3.31–8.63) | <0.01 | 3.96 (2.28–6.87) | <0.01 |
| Hyperthyroidism (oral treatment) | 3.10 (1.26–7.61) | <0.05 | 2.75 (0.99–7.60) | 0.05 |
| DM during pregnancy | 7.12 (4.54–11.20) | <0.01 | 5.73 (3.44–9.55) | <0.01 |
| Autoimmune disease | 1.11 (0.48–2.58) | 0.81 | 0.80 (0.25-2.57) | 0.70 |
| FGR based on the JSOG definition | 1.76 (0.79–3.92) | 0.16 | 1.98 (0.86–4.53) | 0.11 |

b) Odds ratios for CAPO based on the JSOG definition of FGR at 28 weeks of gestation (period 2)

|  | cOR (95% CI) | P | aOR (95% CI) | P |
| --- | --- | --- | --- | --- |
| Prepregnancy BMI <18.5 mg/kg^2^ | 1.57 (1.15–2.16) | <0.01 | 1.84 (1.33–2.55) | <0.01 |
| Smoking status | 1.89 (1.07–3.35) | <0.05 | 1.52 (0.80–2.87) | 0.19 |
| CH | 5.00 (3.03–28.25） | ＜0.01 | 3.86 (2.20–6.79) | <0.01 |
| DM during pregnancy | 7.24 (4.76–12.7) | <0.01 | 5.91 (3.52–9.94) | <0.01 |
| GDM | 1.56 (1.00–2.42) | <0.05 | 1.80 (1.13–2.86) | <0.05 |
| HDP without CH | 1.84 (1.26–2.70) | <0.01 | 1.60 (1.06–2.43) | <0.05 |
| FGR based on the JSOG definition | 4.56 (2.41–8.60) | <0.01 | 3.94 (2.00–7.74) | <0.01 |

c) Odds ratios for CAPO based on the SMFM definition of FGR at 18 weeks of gestation (period 1)

|  | cOR (95% CI) | *P* | aOR (95% CI) | *P* |
| --- | --- | --- | --- | --- |
| Prepregnancy BMI <18.5 kg/m^2^ | 0.73 (0.49–1.11) | 0.14 | 0.84 (0.56–1.27) | 0.41 |
| Smoking during pregnancy | 1.90 (1.09–3.31) | <0.05 | 1.57 (0.83–2.95) | 0.16 |
| Use of assisted reproductive technology | 1.25 (0.91–1.71) | 0.18 | 1.26 (0.88–1.81) | 0.21 |
| CH | 5.35 (3.31–8.63) | <0.01 | 3.96 (2.28–6.86) | <0.01 |
| Hyperthyroidism (oral treatment) | 3.10 (1.26–7.61) | <0.05 | 2.78 (1.01–7.70) | <0.05 |
| DM during pregnancy | 7.12 (4.54–11.20) | <0.01 | 5.64 (3.38–9.39) | <0.01 |
| Autoimmune disease | 1.11 (0.48–2.58) | 0.81 | 0.81 (0.25-2.60) | 0.72 |
| FGR based on the SMFM definition | 2.02 (1.21–3.37) | <0.01 | 1.76 (0.98–3.17) | 0.06 |

d) Odds ratios for CAPO based on the SMFM definition of FGR at 28 weeks of gestation (period 2)

|  | cOR (95% CI) | *P* | aOR (95% CI) | *P* |
| --- | --- | --- | --- | --- |
| Prepregnancy BMI <18.5 mg/kg^2^ | 1.57 (1.15–2.16) | <0.01 | 1.72 (1.25–2.39) | <0.01 |
| Smoking status | 1.89 (1.07–3.35) | <0.05 | 1.42 (0.76–2.64) | 0.27 |
| CH | 5.00 (3.03–28.25） | ＜0.01 | 4.13 (2.42–7.06) | <0.01 |
| DM during pregnancy | 7.24 (4.76–12.7) | <0.01 | 6.39 (3.92–10.4) | <0.01 |
| GDM | 1.56 (1.00–2.42) | <0.05 | 1.72 (1.10–2.71) | <0.05 |
| HDP without CH | 1.84 (1.26–2.70) | <0.01 | 1.69 (1.13–2.52) | <0.05 |
| FGR based on the SMFM definition | 2.85 (1.74–4.69) | <0.01 | 2.60 (1.54–4.39) | <0.01 |

aOR, adjusted odds ratio; BMI, body mass index; CAPO, composite adverse perinatal outcome; CH, chronic hypertension; CI, confidence interval; cOR, crude odds ratio; DM, diabetes mellitus; FGR, fetal growth restriction; GDM, gestational diabetes mellitus; HDP, hypertensive disorders of pregnancy; JSOG, Japan Society of Obstetrics and Gynecology.
